# Botulinum toxin in high-risk BPH patients in retention

**Published:** 2008

**Authors:** Sreedhar Reddy, Arun Chawla, Joseph Thomas

**Affiliations:** Department of Urology, Kasturba Medical College, Manipal, India. E-mail: urologyreddy@yahoo.com

## SUMMARY

This is a prospective study evaluating the efficacy of botulinum toxin A injection in benign prostatic hyperplasia (BPH) patients. Twenty-one men with benign prostatic enlargement on chronic indwelling catheter for at least 3 months who were not candidates for surgery because of poor general condition received 200 U botulinum toxin A in the transition zone by transrectal approach under ultrasound guidance. This was done as an outpatient procedure without any anesthesia. Mean patient age was 80 ± 2 years. No significant local effects occurred. Patients were reevaluated at 1 and 3 months post-treatment. Baseline prostate volume of 70 ± 10 ml decreased to 57 ± 10 ml at 1 month and to 47 ± 7 ml at 3 months. At 1 month, 16 patients (76%) could resume voiding with a mean Qmax OF 9.0 ± 1.2 ml/s. At 3 months, 17 patients (81%) voided with a mean Qmax of 10.3 ± 1.4 ml/s. Residual urine was 80 ± 19 ml and 92 ± 24 ml at 1 and 3 months, respectively. Mean serum total PSA decreased from 6.0 ± 1.1 ng/ml at baseline to 5.0 ± 0.9 ng/ml at 3 months. The authors conclude that botulinum A injection into the prostate swiftly reduces prostate volume and may be a promising treatment for refractory urinary retention in patients with benign prostatic enlargement who are unfit for surgery.

## COMMENTS

The effects of botulinum toxin A on the prostate has been studied in different species, including rats, dogs, and humans.[1] The exact mechanism of action of botulnum toxin A in the prostate is not known. It affects both the static and dynamic components of BPH obstruction. It induces diffuse apoptosis in the prostate gland by inhibiting ACh release and counters sympathetic activity by inhibiting norepinephrine release. Intraprostatic injection is a simple, inexpensive, and minimally invasive treatment option for BPH patients. Recent studies have shown promising results for botulinum toxin A therapy in BPH management, even in small prostates and these effects were sustained at 6- to 12-month follow-up.[2] But all these studies lack in patient number and study design. Properly conducted randomized controlled trials with large case numbers and longer follow-up are required to validate the results obtained in the smaller studies, before botulinum toxin can be incorporated in the treatment armamentarium of BPH. The present study, though not a placebo-controlled study, has to be appreciated in that it has been conducted in patients who are condemned to permanent catheter, but ambulant. Ethical committee approval and patient approval is easier to get in this subset of patients. Till we get a final answer from large randomized controlled trials, botulinum toxin A injection therapy can be tried in high-risk BPH patients with refractory urinary retention, where pharmacotherapy has failed and any type of anesthesia is highly risky.
